# Supplementary material for: Route of Sensitization to Peanut Influences Immune Cell Recruitment at Various Mucosal Sites in Mouse: An Integrative Analysis
Source: Nutrients. 2022 Feb 14;14(4):790. doi: 10.3390/nu14040790 (PMC8875549; doi:10.3390/nu14040790)
Supplement: Supplementary file 1 [file nutrients-14-00790-s001.zip › nutrients-1571132-supplementary.pdf]

Supplementary materials

1. Cellular suspension preparations

All reagents were from Sigma-Aldrich (Saint-Louis, MO, USA) unless stated.

Blood samples: blood was collected by intra-cardiac punction using a 1 mL syringe containing EDTA. After centrifugation, plasma was collected and stored at -20°C until assay.

Ears: Ears were rinsed in PBS and placed in PBS containing 5 mg/mL dispase overnight at 4°C. Tissue were then cut into small pieces and immersed during 30 min at room temperature in trypsin 0.5%, EDTA 0.5 M, containing collagenase 1 mg/mL and DNAse 10 IU/mL (Invitrogen, Carslbad, CA, USA). Dissociated tissue was then passed through a 40 µm cell strainer rinsed by 2 mL of PBS, SVF 2%, HEPES 15 mM (Buffer 1). After centrifugation (300x*g*, 10 min, 4°C) the cell pellets were suspended with 2 mL of FACS buffer (PBS, EDTA 1mM, SVF 2%, sodium azide 0.1%) and kept on ice until labelling.

Broncho-alveolar lavages (BAL): BAL were collected with a 1mL syringe by injecting the lungs with PBS (2 x 1 mL). After centrifugation (500x*g*, 10 min, 4°C), supernatants were collected, aliquoted, and stored at -80°C until cytokine analysis. Cell pellets were kept in FACS buffer on ice until labelling.

Spleen: spleen was mechanically dissociated (gentleMACS™ apparatus and tubes, Miltenyi Biotec) then passed through a 70 µm cell strainer and finally washed in RPMI. After centrifugation (300x*g*, 10 min, 20°C), cell pellets were incubated for 1 min in 1mL of Red Blood Cell Lysing Buffer (Hybri-Max™). After washing in RPMI, cell pellets were kept in FACS buffer on ice until labelling.

Mediastinal Lymph Nodes (MedLN): MedLN were rinsed in PBS and then transferred in digestion buffer (RPMI, collagenase 0.5 mg/mL, DNase 25 IU/mL; 15 min, 37°C). After two mechanical dissociations, they were passed through a cell strainer 70 µm and washed in RPMI. After a first centrifugation (300x*g*, 10 min, 4°C), cell pellets were incubated for 1 min with 1mL of Red Blood Cell Lysing Buffer. Cells were washed in RPMI and then kept in FACS buffer on ice until labelling.

Mesenteric Lymph Nodes (MLN): MLN were rinsed in PBS, transferred in RPMI, HEPES 15mM, collagenase 0.5 mg/mL, DNase 10 IU/mL and incubated for 15 min at 37°C. They were mechanically dissociated, passed through a cell strainer (100 µm) and extensively rinsed in RPMI. After centrifugation (300x*g*, 10 min, 4°C), cell pellets were kept in FACS buffer.

Lungs: lungs were transferred in the same digestion buffer as MLN, and then followed the same treatment as the MedLN.

Intraepithelial lymphocytes (iEL) and lamina Propria (LP): Immediately after collection, intestine was washed with 10 mL of cold PBS. Then Peyer's patches (PP) were removed and intestine was longitudinally opened and cut into small pieces. After two lavages with Buffer 1 on a 100 µm cell strainer, the tissue were collected and incubated for 10 min at 37°C in PBS, SVF 10%, EDTA 5 mM, HEPES 15 mM, penicillin 100 IU / streptomycin 100 µg/mL, and then filtered and rinsed with the same buffer. The collected cellular fraction was centrifuged (300x*g*, 10min, 4°C) and the pellet kept in FACS buffer (iEL). The pieces of intestines were then washed in RPMI, HEPES 15 mM and incubated (30 min, 37°C) in the same buffer as MLN before mechanical dissociation. After filtration on 40 µm cell strainer and centrifugation (300x*g*, 10 min, 4°C), cell pellets were kept in FACS buffer (LP).

2. Immunophenotyping by flow cytometry

After numeration, samples were centrifuged and suspended in FACS buffer containing FcγR Blocking Reagent (clone 2.4G2, BD Biosciences, Le Pont de Claix, France; 1µg/10^6 cells). Extra-cellular staining was performed by incubation for 45 min, +4°C with pre-optimised panel antibodies, all from BioLegend (San Diego, United States; **Error!**

Reference source not found. Table S1) and all used at pre-optimised concentrations (from 0.5 to 2 µl / 10^6 cells). Flow cytometry panels below were used for analysis of inflammatory cells in BAL, lungs, LP and ears (panel A); analysis of dendritic cells subtypes in lungs, spleen, MedLN, LP and MLN (panel B); analysis of T cells and their homing receptors in lungs, ears, LP and BAL; analysis of ILC and NK cells in lungs, iEL, ears and LP. For ILC analysis, lineage (Lin) corresponds to a mix of anti-CD11c (clone N418), anti-F4/80 (clone BM8), anti-erythroid cells (clone TER119), anti-FcεRIα (clone MAR-1), anti-CD3ε (clone 17A2) and anti-CD19 (clone MB19-1). Compensations were performed thanks to UltraComp eBeads (Invitrogen, Thermo Fisher Scientific). Unstained and mono-stained samples were performed by pooling all samples for each organ and each panel.

Stained cells were centrifuged (500x*g*, 6 min, 4°C) and suspended in CytoFix (BD Biosciences) before flow cytometry data acquisition.

Table S1. Flow cytometry panel: targets, clones and fluorochromes used for analysis of **A**. inflammatory cells; **B**. dendritic cells; **C**. T cells and their homing receptors; **D**. ILC and NK cells. *APC: Allophycocyanin; BV: Brillant-Violet; FITC: Fluorescein; PE: Phycoerythrine*

PANELS											
A			B			C			D		
Targets	Clones	Fluorochromes	Targets	Clones	Fluorochromes	Targets	Clones	Fluorochromes	Targets	Clones	Fluorochromes
CD3	*17A2*	FITC	CD3	*17A2*	FITC	CD3	*17A2*	FITC	Mix Lin		FITC
CCR3	*J073E5*	PE	CD103	*2.00E+07*	PE	CCR7	*4B12*	PE	CD49b	*DX5*	PE
CD25	*PC61*	PE-Cy7	MHCII	*M5/114.15.2*	PE-texas red	CCR2	*SA203G11*	Pe-Cy7	CD25	*PC61*	PE-Dazzle 594
Siglec F/ CD170	*S17007L*	Alexa Fluor 647	CCR2	*SA203G11*	Pe-Cy7	CD25	*PC61*	Alexa Fluor 647	CD117	*2B8*	Pe-Cy7
Ly6C	*HK1.4*	BV-421	CD11b	*M1/70*	Alexa Fluor 647	CD8	*53-6.7*	AF700	IL-33Rα (IL1RL1, ST2)	*DIH9*	APC
CD45	*30-F11*	BV-510	CD8	*53-6.7*	AF700	CCR4	*2G12*	BV-421	CD45	*30-F11*	APC-fire 750
CD4	*GK1.5*	BV-605	Siglec H	*551*	Pacific Blue	CD45	*30-F11*	BV-510	CD196 (CCR6)	*29-2L17*	BV-421
F4/80	*BM8*	BV-711	CD45	*30-F11*	BV-510	CD4	*GK1.5*	BV-605	CD4	*RM4-5*	BV-510
Ly6G	*1A8*	BV785	CD4	*GK1.5*	BV-605	CCR8	SA214G2	BV-711	CD335 (NKp46)	*29A1.4*	BV-605
			CD11c	*N418*	BV-711				CD127	*A7R34*	BV-711
									NK-1.1	*PK136*	BV-784

In all analysis, exclusion of debris and then gating of single cells were first performed. Analysis of inflammatory cells was performed within gated $CD45^+CD3^-$ cells: neutrophils were identified as $Ly6C^+Ly6G^+$ cells, eosinophils as $SiglecF^+CCR3^+$ cells and macrophages as $Ly6C^-F4/80^+$ cells (Not shown; panel A). $CD4^+CD8^-$ and $CD8^+CD4^-$ T cells populations were identified within $CD45^+CD3^+$ cells. Within $CD4^+$ or $CD8^+$ T cells, we analysed each homing receptor independently (CCR2, CCR4, CCR7 and CCR8) (figure S1; panel C). Activated T cells were identified as $CD4^+$ $CD25^+$ within $CD45^+$ $CD3^+$ cells (Not shown).

Dendritic cells (DCs, panel B) were defined within $CD45^+CD3^-$ gated cells as $CD11c^+MHCII^+$ cells. Within DC, we identified plasmacytoid dendritic cells (pDCs, $SiglecH^+$), conventional DCs (cDC) 1 ($CD8^+CD11b^-$) and cDC2 ($CD11b^+CD8^-$) in spleen, MedLN and MLN (as exemplified for spleen in figure S2). For lungs and LP tissue, we analysed DC subtypes based on CD11b and CD103 expression, and pDCs were identi-

fied thanks to SiglecH expression within $CD11b^-$ $CD103^-$ cells (as exemplified for lung in figure S3).

For Innate lymphoid cells (ILCs, panel D), we first select the Lin-$CD45^+$ cells, in which ILCs were defined as $CD127^+$ within $CD4^-$ cells. ILC subtypes were defined according to expression of ST2 (ILC2), NK1.1 (ILC1), NKp46 / CCR6 (ILC3 NCR^- /LTi or ILC3 NCR^+). Natural killer (NK) cells were defined within $CD45^+CD127^-$ as $NKp46^+NK1.1^+$ cells (as exemplified in figure S4).

The different cells analysed and available for each tissue/lymph node are detailed below.

Lungs: $CD45^+$, T lymphocytes, Eosinophils, Macrophages, Neutrophils, $CD4^+$, $CD8^+$, $CCR2^+$/$CCR4^+$/$CCR7^+$/$CCR8^+$ within $CD4^+$ or $CD8^+$, $CD4^+CD25^+$, ILC, ILC1, ILC2, ILC3, NK, DC $CD11c^+MHCII^+$, DC $CD11b^+CD103^+$, DC $CD11b^+CD103^-$, DC $CD11b^-$ $CD103^+$, pDC SiglecH+

BAL: CD45, T lymphocytes, Eosinophils, Macrophages, Neutrophils, $CD4^+$, $CD8^+$, $CCR2^+$/$CCR4^+$/$CCR7^+$/$CCR8^+$ within $CD4^+$ or $CD8^+$, $CD4^+CD25^+$

LP: $CD45^+$, T lymphocytes, Eosinophils, Macrophages, Neutrophils, $CD4^+$, $CD8^+$, $CCR2^+$/$CCR4^+$/$CCR7^+$/$CCR8^+$ within $CD4^+$ or $CD8^+$, ILC, ILC1, ILC2, ILC3, NK, DC $CD11c^+MHCII^+$, DC $CD11b^+CD103^+$, DC $CD11b^+CD103^-$, DC $CD11b^-CD103^+$, pDC $SiglecH^+$

iEL: ILC1, ILC2, ILC3, NK

Ears: $CD45^+$, T lymphocytes, Eosinophils, Macrophages, Neutrophils, $CD4^+$, $CD4^+CD25^+$, $CD8^+$

Spleen, MLN and MedLN: DC, cDC1, cDC2, pDC

3. Results

3.1. Variable Importance in the Projection (VIP)

VIP values were calculated by PLS-DA modelling of all the immune cellular components, with the exposure route as the explanatory variable (Table S2). Variables were considered discriminant for VIP +/- SD values > 1.

Table S2. VIP values issued from supervised analysis of immune cellular components using PLS-DA modelling. **A** correspond to the beginning of the table and **B** the ending part. LP: lamina Propria; MLN: Mesenteric lymph node; iEL: Intraepithelial lymphocytes; MedLN: Mediastinal Lymph Node; BAL: Broncho-alveolar lavage; NK: Natural Killer; ILC: Innate lymphoid cell

**A**	Axis 1			Axis 2		
Variable	VIP	SD	VIP-SD	VIP	SD	VIP-SD
BAL CCR2+ within CD4	1.866	0.130	1.736	1.438	0.080	1.358
BAL CCR4+ within CD4	1.855	0.132	1.723	1.442	0.080	1.362
BAL CCR8+ within CD4	1.856	0.134	1.721	1.441	0.081	1.360
BAL CCR7+ within CD4	1.854	0.134	1.720	1.442	0.079	1.363
BAL CD4+	1.854	0.136	1.717	1.439	0.083	1.356
BAL CCR2+ within CD8	1.848	0.138	1.710	1.389	0.075	1.314
BAL CCR4+ within CD8	1.841	0.142	1.699	1.384	0.077	1.306
BAL CCR8+ within CD8	1.836	0.146	1.689	1.380	0.081	1.299
BAL CCR7+ within CD8	1.828	0.139	1.688	1.374	0.075	1.298
BAL CD8+	1.832	0.147	1.685	1.377	0.081	1.296
BAL CD45	1.720	0.118	1.602	1.336	0.089	1.247
BAL CD4+CD25+	1.752	0.160	1.592	1.387	0.092	1.295
Lung pDC SiglecH+	1.725	0.150	1.576	1.359	0.075	1.284
Lung CD11b+CD103-	1.693	0.241	1.452	1.378	0.139	1.239
BAL Macrophages	1.594	0.169	1.424	1.229	0.155	1.074
Spleen DC CD11c+CMHII+	1.599	0.177	1.422	1.466	0.126	1.340
BAL Neutrophils	1.571	0.161	1.409	1.198	0.142	1.056
Lung ILC3	1.497	0.125	1.372	1.108	0.127	0.981
BAL LyT	1.559	0.188	1.371	1.253	0.156	1.098
Lung ILC	1.457	0.137	1.320	1.081	0.123	0.957
BAL Eosino	1.505	0.209	1.296	1.203	0.176	1.027
Lung CD11b-CD103+	1.498	0.210	1.288	1.123	0.161	0.962
Spleen cDC2 CD11b+CD8-	1.273	0.276	0.997	1.007	0.206	0.800
MedLN cDC1 CD8+CD11b-	1.083	0.154	0.930	1.108	0.212	0.896
Lung CCR2+ within CD4	1.145	0.239	0.906	1.022	0.245	0.777
LP "NK"	1.160	0.294	0.866	0.863	0.266	0.597
Lung CD4+CD25+	1.161	0.315	0.846	1.284	0.191	1.093
Lung CCR8+ within CD4	1.016	0.286	0.729	1.158	0.309	0.849
LP ILC3	0.920	0.231	0.689	0.796	0.567	0.230
LP ILC	0.901	0.229	0.672	0.842	0.600	0.242
Lung ILC1	0.842	0.192	0.650	0.623	0.357	0.266
Lung CCR7+ within CD4	0.898	0.259	0.639	1.053	0.303	0.751
Lung ILC2	0.876	0.246	0.630	0.751	0.180	0.571
Lung CD11b+CD103+	0.884	0.272	0.612	0.733	0.325	0.408
Lung CCR4+ within CD4	0.893	0.318	0.575	1.146	0.364	0.782
Ear Eosino	0.800	0.246	0.554	0.866	0.393	0.472
MedLN pDC SiglecH+	0.751	0.216	0.535	1.190	0.247	0.942
Lung "NK"	0.796	0.278	0.518	0.874	0.246	0.628
iEL ILC2	0.830	0.320	0.510	1.372	0.250	1.122
Ear CD8+	0.702	0.219	0.484	0.743	0.335	0.408
LP CD11b-CD103+ | Freq. of CD45+ tot	0.743	0.289	0.455	0.892	0.280	0.612
Lung LyT	0.799	0.348	0.451	1.103	0.590	0.513
Lung DC CD11c+CMHII+	0.753	0.311	0.442	0.664	0.331	0.333
Ear Macrophages	0.665	0.238	0.426	0.865	0.402	0.463
Ear CD45+	0.648	0.259	0.389	1.006	0.446	0.560

Ear LyT	0.645	0.258	0.388	0.976	0.442	0.534
LP CD11b+CD103-	0.630	0.255	0.374	1.040	0.333	0.708

**B**	Axis 1			Axis 2		
Variable	VIP	SD	VIP-SD	VIP	SD	VIP-SD
LP ILC1	0.772	0.429	0.343	1.055	0.446	0.610
Lung CCR2+ within CD8+	0.788	0.463	0.325	0.776	0.207	0.570
Lung CD45+	0.638	0.328	0.310	1.106	0.607	0.499
LP CCR4+ within CD4	0.513	0.244	0.269	0.563	0.315	0.249
Lung CCR8+ within CD8+	0.761	0.514	0.247	0.926	0.230	0.696
Lung Eosinophils	0.472	0.229	0.243	0.502	0.602	-0.100
LP CCR7+ within CD4	0.557	0.323	0.235	0.596	0.376	0.219
Ear CD4+CD25+	0.459	0.232	0.227	0.357	0.173	0.184
LP CCR8+ within CD4	0.483	0.271	0.212	0.477	0.339	0.138
LP ILC2	0.480	0.268	0.212	1.260	0.488	0.773
Lung CD4+	0.570	0.364	0.205	1.295	0.353	0.942
Lung Neutrophils	0.479	0.276	0.202	1.123	0.380	0.743
LP pDC SiglecH+	0.403	0.207	0.197	0.432	0.256	0.177
Lung Macrophages	0.461	0.280	0.181	1.147	0.362	0.785
Lung CCR7+ within CD8+	0.678	0.503	0.175	0.918	0.251	0.667
LP CD4+	0.440	0.306	0.134	0.617	0.416	0.201
iEL ILC	0.514	0.407	0.107	1.286	0.357	0.929
Spleen pDC SiglecH+	0.333	0.241	0.091	1.389	0.338	1.051
LP DC CD11c+CMHII+	0.349	0.272	0.077	0.482	0.490	-0.008
Lung CCR4+ within CD8+	0.625	0.561	0.064	0.902	0.283	0.619
Spleen cDC1 CD8+CD11b-	0.422	0.358	0.064	0.468	0.314	0.154
Lung CD8+	0.556	0.498	0.058	1.107	0.262	0.845
MLN cDC1 CD8+CD11b-	0.461	0.424	0.037	0.347	0.417	-0.070
iEL ILC3	0.415	0.416	-0.001	1.138	0.378	0.760
Ear Neutrophils	0.209	0.252	-0.042	1.149	0.289	0.860
Ear CD4+	0.299	0.352	-0.052	0.268	0.300	-0.032
LP CD45+	0.497	0.551	-0.053	1.000	0.259	0.741
LP CCR2+ within CD8	0.393	0.457	-0.064	0.990	0.388	0.602
MLN pDC SiglecH+	0.194	0.265	-0.071	0.231	0.436	-0.206
LP LyT	0.410	0.488	-0.078	0.950	0.280	0.670
LP CCR2+ within CD4	0.224	0.308	-0.084	0.652	0.468	0.184
MLN DC CD11c+CMHII+	0.302	0.387	-0.085	0.456	0.237	0.219
LP Macrophages	0.309	0.395	-0.086	0.229	0.346	-0.117
LP CD4+CD25+	0.197	0.286	-0.089	0.516	0.431	0.085
MLN cDC2 CD11b+CD8-	0.321	0.415	-0.094	0.377	0.256	0.121
LP Neutrophils	0.353	0.468	-0.115	0.707	0.325	0.382
MedLN cDC2 CD11b+CD8-	0.044	0.173	-0.129	0.638	0.450	0.188
LP CCR4+ within CD8	0.266	0.421	-0.155	0.656	0.368	0.288
iEL ILC1	0.039	0.201	-0.162	0.636	0.473	0.163
MedLN DC CD11c+CMHII+	0.054	0.229	-0.175	0.925	0.291	0.634
LP CD11b+CD103+	0.062	0.243	-0.181	0.347	0.452	-0.105
LP Eosinophils	0.018	0.271	-0.253	0.128	0.271	-0.143
iEL "NK"	0.025	0.294	-0.269	1.115	0.492	0.622
LP CD8+	0.060	0.440	-0.380	0.607	0.561	0.046
LP CCR7+ within CD8	0.035	0.435	-0.401	0.526	0.565	-0.039
LP CCR8+ within CD8	0.008	0.461	-0.453	0.341	0.558	-0.217

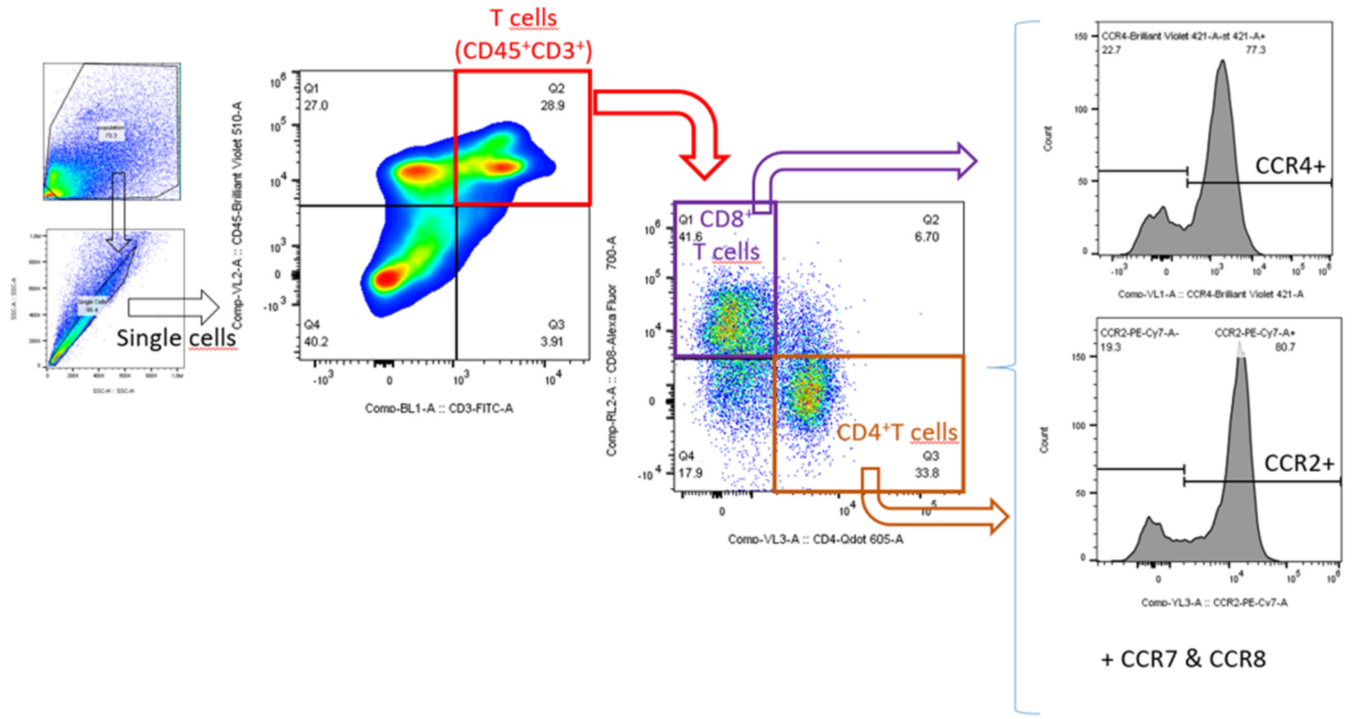

Figure S1: Gating strategy to analyse T cells sub-populations.

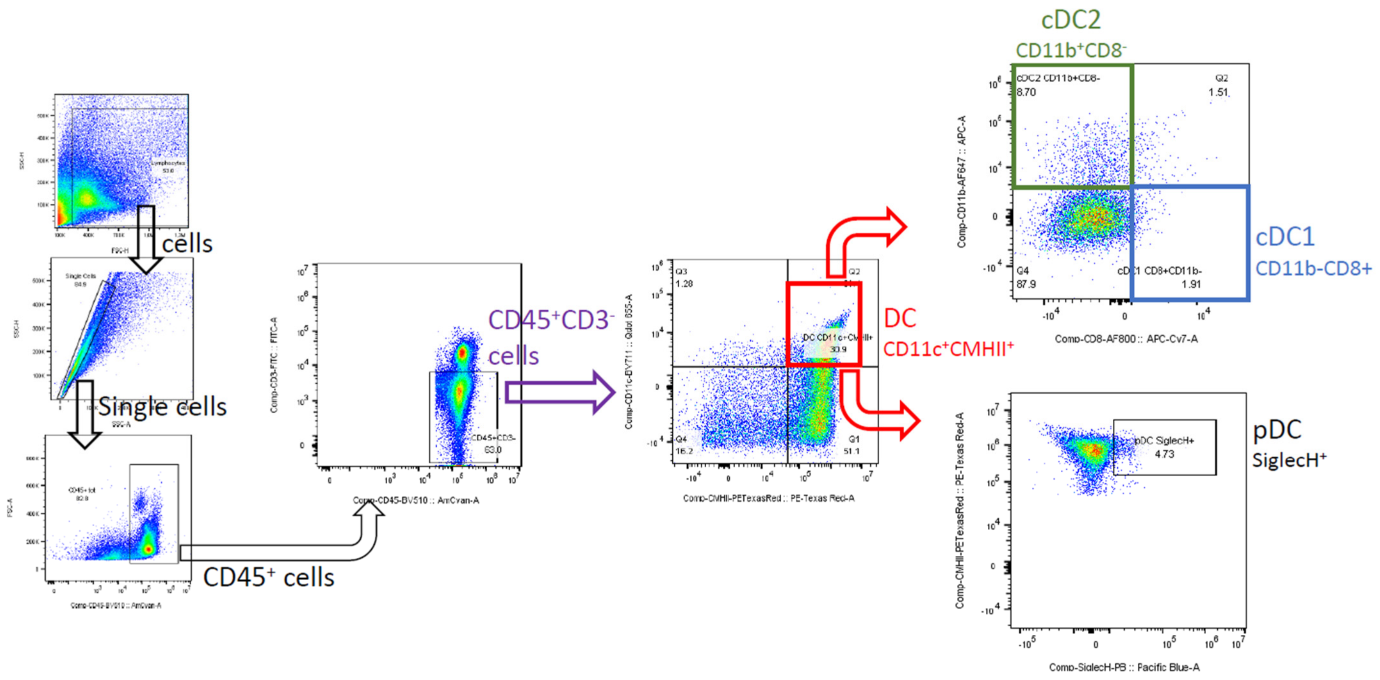

Figure S2. Gating strategy to analyse dendritic cells (DCs) sub-populations from lymphoid tissue (spleen, Mesenteric lymph node, Mediastinal Lymph Node). Analysis of cells from spleen is shown. cDC: conventional DC; pDC: plasmacytoid DC

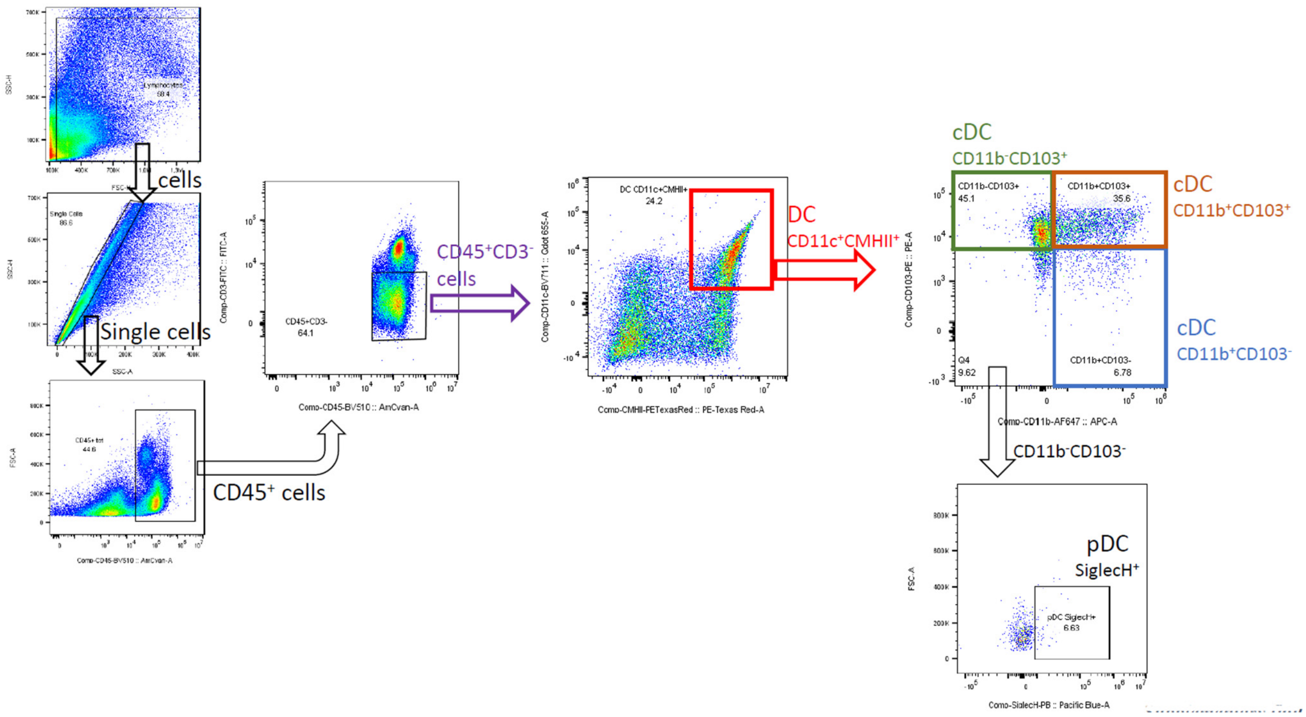

Figure S3. Gating strategy to analyse Dendritic cells (DC) sub-populations from lungs, ears, lamina propria and broncho-alveolar lavage. Analysis of cells extracted from lung is shown. cDC: conventional DC; pDC: plasmacytoid DC

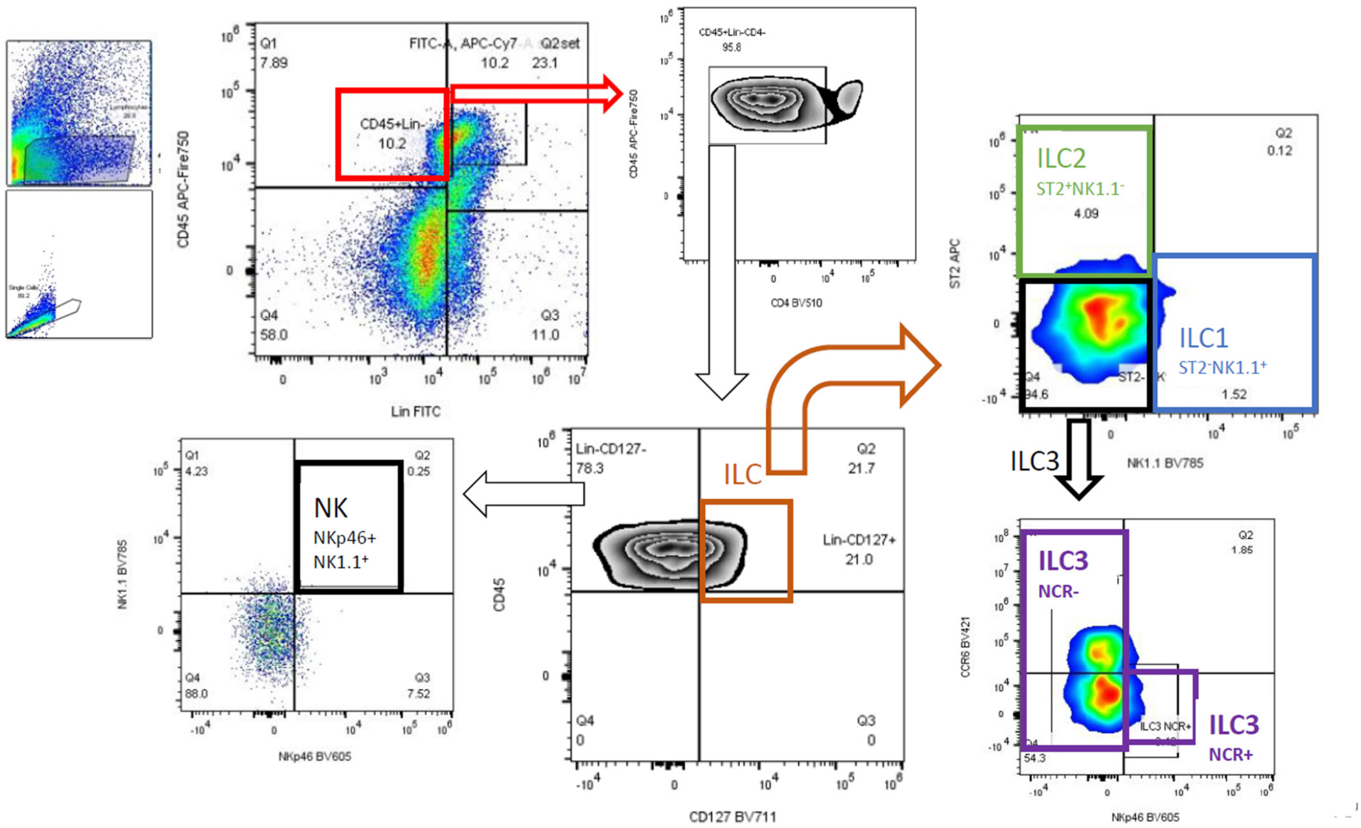

Figure S4. Gating strategy to analyse innate lymphoid and Natural Killer cells in cell extracted from lungs, intraepithelial lymphocytes, ears and lamina propria (LP). Analysis of cells extracted LP is shown.

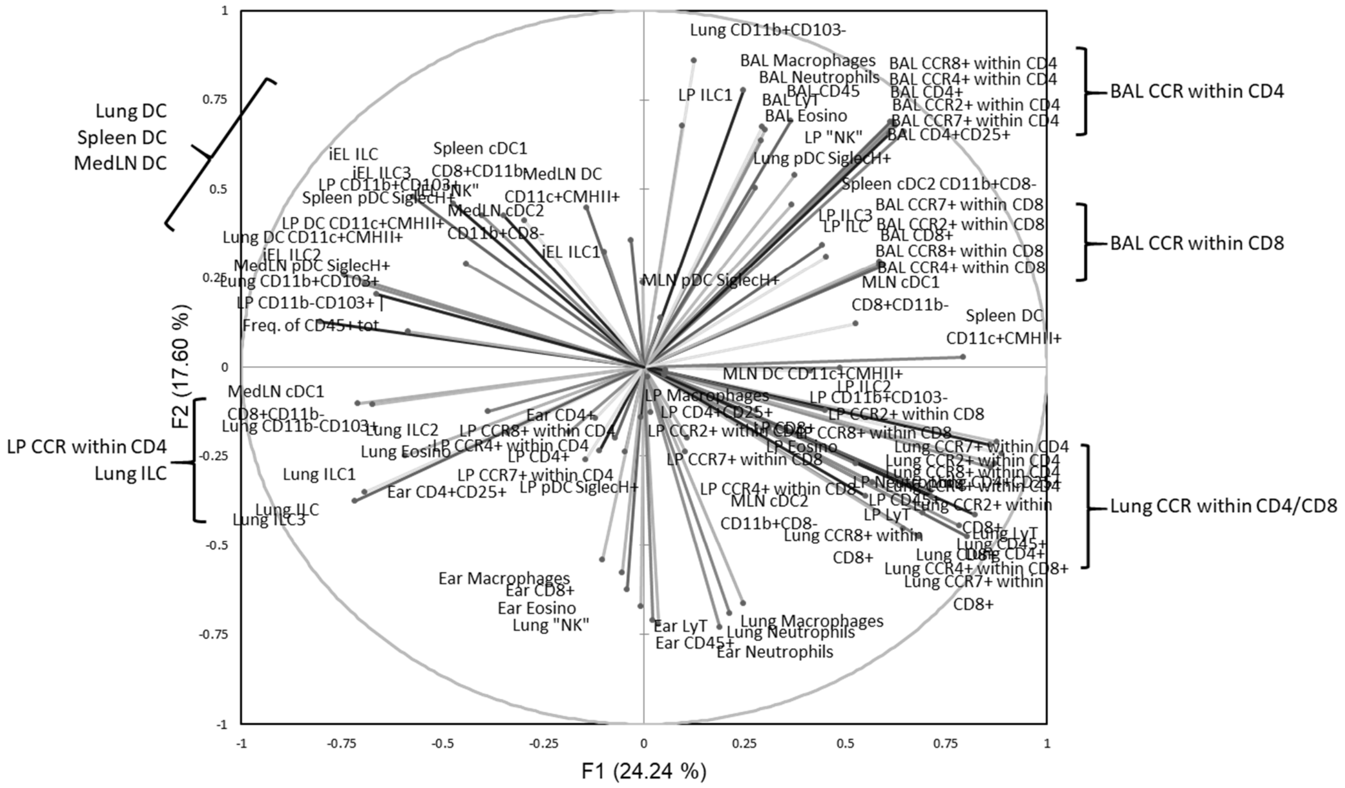

Figure S5. Loading plots from PCA on immune cellular components in tissues, lymph nodes and broncho-alveolar lavage fluids. ILC: innate lymphoid cells; NK: natural killer

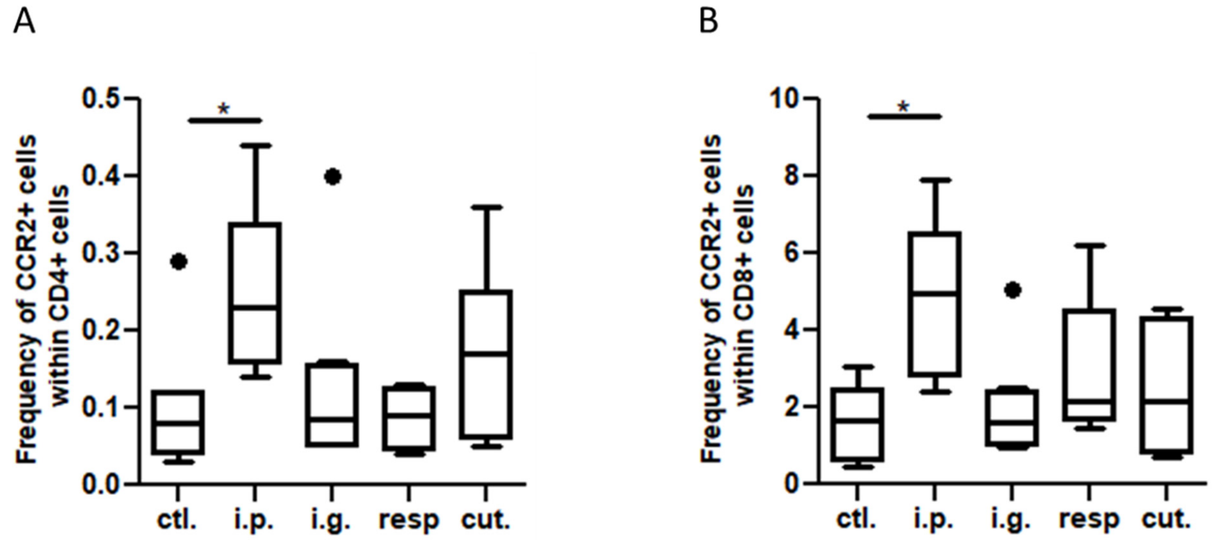

Figure S6. T cells influx in lamina propria (LP): frequencies of CCR2$^+$ within CD4$^+$ (**A**) and within CD8$^+$ (**B**) in LP from mice exposed through the intra-peritoneal (i.p., *n* =8), intra-gastric (i.g., *n* =8), respiratory (resp, *n* = 5) or cutaneous (cut., *n* = 7) routes. * p <0.05: Using non-parametric Kruskal Wallis test and Dunn's post-test when comparing all groups to control group (non-exposed mice). Bold black dots represent outliers.

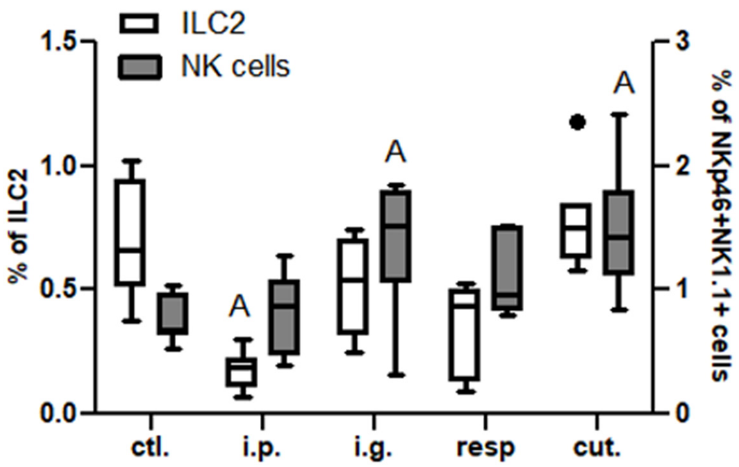

Figure S7. Innate lymphoid cells within intraepithelial lymphocytes (iEL): ILC2 ($ST2^+NK1.1^+$; empty bars) and Natural Killer cells ($NK1.1^+NKp46^+$ cells; grey bars) frequencies within iEL from mice exposed through the different routes. Frequencies are expressed using $CD45^+$ cells as a reference. "A": indicates a significant difference using non-parametric Kruskal Wallis test and Dunn's post-test when comparing all groups to control one (non-exposed mice). The bold black dot represents an outlier.

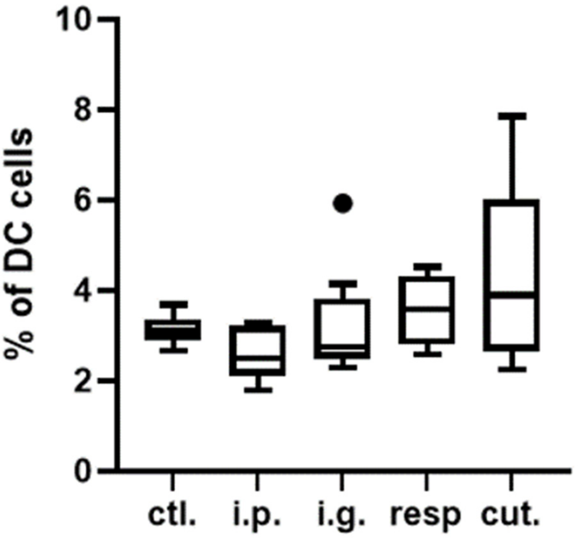

Figure S8. Dendritic cells in mediastinal lymph node in the different groups of exposed mice. The bold black dot represents an outlier.
